# Safety and Immunogenicity of a Novel Intranasal Influenza Vaccine (NasoVAX): A Phase 2 Randomized, Controlled Trial

**DOI:** 10.3390/vaccines9030224

**Published:** 2021-03-05

**Authors:** Sybil Tasker, Anna Wight O’Rourke, Anvar Suyundikov, Peta-Gay Jackson Booth, Stephan Bart, Vyjayanthi Krishnan, Jianfeng Zhang, Katie J. Anderson, Bertrand Georges, M. Scot Roberts

**Affiliations:** 1 Altimmune, Inc., Gaithersburg, MD 20878, USA; sybil.tasker@me.com (S.T.); 1217anna@gmail.com (A.W.O.); asuyundikov@altimmune.com (A.S.); vkrishnan@altimmune.com (V.K.); jzhang@altimmune.com (J.Z.); katieanderson1@gmail.com (K.J.A.); bgeorges@altimmune.com (B.G.); 2 Codagenix, Inc., Farmingdale, NY 11735, USA; 3 Biomedical Advanced Research and Development Authority, Washington, DC 20201, USA; 4 Optimal Research, LLC, Rockville, MD 20850, USA; pgjacksonbooth@optimalsites.net (P.-G.J.B.); sbart@optimalsites.net (S.B.); 5 Vaccitech Limited, Oxford OX4 4GE, UK

**Keywords:** influenza vaccine, intranasal vaccine, adenovirus vector, NasoVAX, mucosal immunity, pre-existing immunity

## Abstract

Annual influenza vaccination greatly reduces morbidity and mortality, but effectiveness remains sub-optimal. Weaknesses of current vaccines include low effectiveness against mismatched strains, lack of mucosal and other effective tissue-resident immune responses, weak cellular immune responses, and insufficiently durable immune responses. The safety and immunogenicity of NasoVAX, a monovalent intranasal influenza vaccine based on a replication-deficient adenovirus type 5 platform, were evaluated in a placebo-controlled single ascending-dose study. Sixty healthy adults (18–49 years) received a single intranasal dose of 1×10^9^ viral particles (vp), 1 × 10^10^ vp, or 1 × 10^11^ vp of NasoVAX or placebo. NasoVAX was well-tolerated and elicited robust influenza-specific systemic and mucosal immune responses. The highest NasoVAX dose and the approved Fluzone^®^ influenza vaccine elicited comparable hemagglutination inhibition (HAI) geometric mean titers (152.8 vs. 293.4) and microneutralization (MN) geometric mean titers (142.5 vs. 162.8), with NasoVAX HAI titers maintained more than 1-year on average following a single dose. Hemagglutinin-specific T cells responses were also documented in peripheral mononuclear cell (PBMC) preparations. Consistent with the intranasal route of administration, NasoVAX elicited antigen-specific mucosal IgA responses in the nasopharyngeal cavity with an increase of approximately 2-fold over baseline GMT at the mid- and high-doses. In summary, NasoVAX appeared safe and elicited a broad immune response, including humoral, cellular, and mucosal immunity, with no impact of baseline anti-adenovirus antibody at the most immunogenic dose.

## 1. Introduction

Influenza is one of the most common viral respiratory infections worldwide, causing 3 to 5 million severe infections and between 290,000 and 650,000 deaths annually [1]. Seasonal influenza vaccination greatly reduces morbidity and mortality, but effectiveness is sub-optimal, averaging about 40% (range 10–60%) over 2004 to 2019 [2].

Most influenza vaccines consist of inactivated split viruses or subunit influenza antigens and are given by injection to elicit serum hemagglutination inhibiting (HAI) antibodies. HAI titer is currently considered as a surrogate marker of activity that is reasonably likely to predict clinical benefit. However, these vaccines are not effective at inducing antiviral T cell responses or mucosal immune responses, two important additional contributors to protection against respiratory viruses [3,4,5,6,7,8]. Furthermore, serum antibody titers wane quickly and may fall below effective levels within influenza season, especially in the elderly [9,10]. Although it is important to continue to use these vaccines, there is consensus that development of improved influenza vaccines is a public health priority. Recently, the National Institute of Allergy and Infectious Diseases (NIAID) presented a strategic plan for development of improved influenza vaccines [11]. That report highlighted several weaknesses of current vaccines, including low effectiveness against mismatch strains, lack of mucosal and other effective tissue-resident immune responses, weak cellular immune responses, and insufficiently durable immune responses.

Replication-deficient adenovirus vectors, especially those based on adenovirus serotype 5 (Ad5), have been evaluated widely in vaccine and gene therapy development programs because they efficiently infect a wide variety of cell types, and a significant body of evidence supports their safety as delivery vectors [12]. However, unmodified adenovirus type 5 is a natural human pathogen responsible for common cold-like symptoms and approximately half of the United States population is seropositive for Ad5. Several studies have indicated that pre-existing immunity to the vector can attenuate its activity (reviewed in [13,14,15]), spurring the development of human adenoviral vector types with lower seroprevalence and nonhuman primate adenoviral vector types. Importantly, unlike injected adenovirus, several preclinical studies have shown that intranasal administration of Ad5 vectors can bypass or overcome pre-existing immunity against the vector [16].

NasoVAX is an intranasal influenza vaccine candidate based on a replication-deficient Ad5 vector platform. NasoVAX is designed to express the hemagglutinin (HA) protein of targeted influenza strains in epithelial cells of the respiratory mucosa to drive local mucosal immunity as well as systemic serum antibody and cellular immune responses. In a clinical study of 5 × 10^8^ viral particles (vp) delivered intranasally, NasoVAX was well-tolerated and able to elicit systemic HAI antibody responses even in the presence of pre-existing Ad5 immunity [17]. The objectives of this study were to evaluate the safety and immunogenicity of NasoVAX in healthy adults (18–49 years) following administration of single intranasal dose. NasoVAX was well-tolerated and elicited robust influenza-specific systemic and mucosal immune responses. Safety and Here we report the safety and immunogenicity. A single intranasal dose of NasoVAX elicited a local, influenza-specific mucosal immunoglobulin A (IgA) response in addition to systemic antibody and T cell responses. Moreover, comparable immune responses were obtained in the absence or presence of pre-existing adenovirus immunity. Finally, NasoVAX induced durable antibody responses that were maintained at seroprotective levels for at least 1-year postvaccination on average.

## 2. Material and Methods

### 2.1. Study Procedures 

This was a Phase 2 randomized, double-blind, placebo-controlled single ascending-dose study conducted between September 2017 and June 2018 at one site in the United States (Optimal Research, LLC, Rockville, MD, USA). The study was designed to evaluate the safety and immunogenicity of NasoVAX in healthy adults 18 to 49 years of age (trial registration at clinicaltrials.gov: NCT03232567 (accessed on 4 Mar 2021)). Subjects were enrolled into 1 of 3 sequential 20-subject cohorts, each defined by vaccine dose (1 × 10^9^, 1 × 10^10^, 1 × 10^11^ vp). Within each dose cohort, subjects were randomized (3:1), based on a computer-generated randomization schedule (block size = 4), to receive a single intranasal dose of NasoVAX or placebo on Day 1.

A safety review committee evaluated the safety of study drug on a blinded basis through Day 29, with safety affirmed on Day 8 for the first 5 subjects in each cohort before the remaining 15 subjects were dosed and for all subjects within a dose cohort before subjects were randomized in the next higher dose cohort.

Approximately 4 months prior to the conduct of this study, a separate study (trial registration at clinicaltrials.gov: NCT03163342 (accessed on 4 Mar 2021)) was conducted to obtain immunogenicity samples from 20 subjects who were administered inactivated quadrivalent influenza vaccine licensed for use in the 2016–2017 influenza season (Fluzone) and containing an A/California/04/2009(H1N1)-like component homologous to the one used for NasoVAX. The Fluzone study was performed at the same investigational site using the same eligibility criteria and schedule of assessments as the NasoVAX study. Immunogenicity samples from the Fluzone study were stored frozen and evaluated for blinded immunogenicity testing in parallel with the samples from the NasoVAX study.

An extension study (trial registration at clinicaltrials.gov: NCT03760549 (accessed on 4 Mar 2021)) was conducted following the conclusion of the NasoVAX trial to evaluate the durability of the HAI response. The additional assessments were obtained from 8 of the 15 subjects receiving the 1 × 10^11^ vp NasoVAX dose and occurred between 10 and 14 months (mean 13.5 months) after vaccination with NasoVAX.

### 2.2. Investigational Product

NasoVAX is based on a E1/E3-deleted, replication-deficient adenovirus serotype 5 vector. The monovalent vaccine used in this study expressed the full-length hemagglutinin (HA) protein from an A/California/04/2009(H1N1)-like influenza strain. NasoVAX was obtained from PER.C6 cells following purification of infected cell lysates and formulation in 10 mM TRIS, 75 mM NaCl, 0.2% PS80, 5% sucrose, 1 mM MgCl2, 0.1 mM EDTA, 0.5% ethanol, and 10 mM L-histidine. (Emergent Manufacturing Operations at Baltimore). NasoVAX was supplied to the site in single-use glass vials, each containing a nominal volume of 0.7 mL of a sterile, frozen suspension of vaccine formulated to deliver the nominal dose. Placebo was normal saline. An unblinded pharmacist at the clinical site prepared the study drug doses in intranasal delivery devices comprised of an LMA MAD300 intranasal mucosal atomization devise (Teleflex) affixed to a 1 mL tuberculin syringe. Other investigational site staff and laboratory staff were blinded to assignment. The devices and contents were identical in appearance for the investigational product and placebo.

Subjects in the inactivated influenza vaccine immunogenicity study received Fluzone Quadrivalent (2016–2017 influenza season, Sanofi Pasteur Inc., Swiftwater, PA, USA). The H1N1 component of Fluzone Quadrivalent contained an influenza A/California/04/2009-like strain homologous to the one expressed by NasoVAX used in this study.

### 2.3. Safety Assessments 

The primary outcome measures were the number of treatment-emergent adverse events (AEs) and the number of treatment-emergent reactogenicity events in study participants. All AEs were recorded until Day 29; thereafter to 6 months postdose only serious adverse events (SAEs), medically attended AEs, and new-onset chronic illnesses were recorded. Each subject recorded specified local and systemic events and oral temperature daily in a paper diary for 14 days postdose.

### 2.4. Immunogenicity Assessments 

Secondary study outcomes were the HAI and microneutralization (MN) immune responses between study Days 1–181. Serum samples from all subjects were assayed for influenza HAI and MN predose and on Days 15, 29, 91, and 181. HAI was expressed as geometric mean titer (GMT; antilog of mean log-transformed titers), geometric mean ratio (GMR; ratio of postvaccination and prevaccination GMTs within the same dose group), seroprotection rate (SPR; percentage of subjects with a HAI titer ≥ 1:40), and seroconversion rate (SCR; percentage of subjects with either a baseline HAI titer < 1:10 and a postvaccination titer ≥ 1:40 or a baseline HAI titer ≥ 1:10 and a 4-fold increase in postvaccination HAI titer relative to baseline). Microneutralization results were expressed as GMT, GMR, and responder rate (the proportion of subjects with 4-fold rise over baseline).

Exploratory endpoints included 1) serum HAI and MN against non-represented influenza strains [A/Brisbane/59/2007(H1N1), A/Saint-Petersburg/61/2015(H1N1), A/New Jersey/8/76(H1N1), A/Perth/16/2009(H3N2), A/Vietnam/1203/2004(H5N1) × A/Puerto Rico/8/34(H1N1)] performed at predose and Day 29; 2) T cell response by ELISpot predose and on Day 8; 3) enzyme-linked immunosorbent assay (ELISA) for influenza A/California/07/2009(H1N1)-specific mucosal IgA performed on the nasopharyngeal swab samples during screening and Day 29, 4) serum Ad5 neutralizing antibody predose and Day 29, and 5) effect of predose Ad5 immunity on measures of NasoVAX immunogenicity. After this study concluded, subjects in the 1 × 10^11^ vp dose group were invited to return for an additional blood sample to evaluate the durability of the HAI response to A/California/07/2009(H1N1) 10 to 14 months (mean 13.5 months) post-vaccination.

### 2.5. Hemagglutination Inhibition Assay (HAI) 

HAI tests were performed by Southern Research Institute (Birmingham, AL) in accordance with validated procedures using horse erythrocytes and influenza virus A/California/07/2009 (H1N1). Following treatment with receptor destroying enzyme, sera were serially diluted 2-fold, starting from 1:10 dilution in a volume of 25 µL/well in 96-well V-bottom plates. Equal volume of virus diluted to 4 HAU/25 µL per well was then added and incubated at room temperature for 30 to 60 min. A 50 µL suspension of horse red blood cells was then added before incubation at room temperature for an additional 60 to 75 min. HAI titer was measured as the reciprocal of the last dilution that was negative for agglutination. A titer of 5 was assigned for negative samples (the reciprocal of half the first dilution). HAI assays were also performed with divergent influenza strains [A/Brisbane/59/2007(H1N1), A/Saint-Petersburg/61/2015(H1N1), A/New Jersey/8/76(H1N1), A/Perth/16/2009(H3N2), A/Vietnam/1203/2004(H5N1) × A/Puerto Rico/8/34(H1N1)] by the method described above.

### 2.6. Microneutralization (MN) Assay

The MN assay was performed according to a validated assay procedure (Southern Research Institute, Birmingham, AL, USA). MN assays were performed using Madin-Darby Canine Kidney (MDCK) cells and wild-type influenza virus [A/California/07/2009 (H1N1)]. In 96-well, flat-bottom plates (50 µL/well), heat-inactivated sera were serially diluted 2-fold, starting from 1:10 dilution. Equal volumes of influenza virus, diluted to 100 tissue culture infectious doses per well, in medium were added, and plates were incubated at 37 °C for 60 to 120 min. After incubation, MDCK cells were added at 1.5 × 10^4^ cells/well in 100 µL of medium, and the plates were further incubated for 19 to 21 h at 37 °C in 5% CO_2_. At that time, cells were fixed with 80% acetone in 1x DPBS for 10 to 15 min at room temperature. After washing, the primary staining antibodies (mouse monoclonal anti-influenza A nucleoprotein antibodies) were added and incubated at room temperature for 60 to 75 min. After additional washing, the secondary staining antibodies (goat anti-mouse IgG conjugated with horseradish peroxidase antibodies) were added and incubated at room temperature for an additional 60 to 75 min. After the final wash, the enzyme substrate was added and incubated at room temperature for 15 to 25 min and the reaction stopped with a stop solution. Absorbance was determined using a spectrophotometer at 490–495 nm, and the 50% virus neutralization (NT) titer of each serum was determined. MN assays were also performed with divergent influenza strains [A/Brisbane/59/2007(H1N1), A/Saint-Petersburg/61/2015(H1N1), A/New Jersey/8/76(H1N1), A/Perth/16/2009(H3N2), A/Vietnam/1203/2004(H5N1) × A/Puerto Rico/8/34(H1N1)] by the method described above.

### 2.7. Hemagglutinin-Specific T Cells by IFN-γ ELISpot

Peripheral blood mononuclear cells (PBMC) were isolated from whole blood by gradient technique using Lymphoprep™ (Axis-Shield, Dundee, UK) within 8 h after venipuncture. After 18 to 72 h at −80 °C, samples were transferred to liquid nitrogen for long-term storage. ELIspot assays were performed using a human IFN-gamma ELISpot Development Module from R&D System (SEL285) following the manufacturer’s recommendations. After thawing, PBMC cell suspensions (150,000 cells/well) were placed in individual wells of PVDF-bottom plates (Millipore, MSIPS4510, Darmstadt, Germany) pre-coated with the human IFN-γ capture antibody. Cells were stimulated with an array of 139 peptides spanning the HA protein of the A/California/04/2009 (H1N1) pdm09 strain of influenza virus (GenPept: ACQ76318) obtained from BEI Resources (NR-15433) and arranged into 7 pools, each containing 19 or 20 peptides, and used at a final concentration of 2 µg/peptide/mL. Following an 18-hour stimulation, plates were incubated with the biotinylated human IFN-γ detection antibody. Signal detection was performed using the ELISpot Blue Color Module from R&D system (SEL 002) following manufacturer’s recommendations. Developed plates were counted with a CTL ImmunoSpot^®^ S5 Reader and spot-forming units (SFU) were measured in each well automatically by the CTL ImmunoSpot^®^ software. After background subtraction, the sum of SFU for the seven HA peptide pools was calculated and expressed as SFU per million PBMC.

### 2.8. HA-Specific Mucosal IgA 

HA-specific IgA antibodies in nasopharyngeal samples were measured using an ELISA assay developed and qualified by Vismederi (Siena, Italia). The nasopharyngeal swabs, one swab per nostril, were collected in individual sample collection tubes without transport media and were stored at −80 °C. A commercial DNA extraction kit (Qiagen, QIASymphony, Hilden, Germany) was used for DNA extraction and purification. For extraction, sample collection tubes were placed on ice while the Spin-X centrifuge tubes were prepared and labelled. For each nasal swab, the swab was removed from the collection tube and inserted into the Spin-X centrifuge tube and 300 L of chilled extraction buffer (0.25M NaCl in PBS with protease inhibitors) was added and vortexed for 1 minute. The Spin-X tube was centrifuged at 13,000 rpm for 15 min at 4 °C. The extraction was repeated by adding additional 300 L of chilled extraction buffer to the top chamber containing the same nasal swab. The extracted samples from both right and left nostril swabs were then pooled in the same tube while on ice and vortexed to mix. Extracted samples were stored at −80 °C freezer until use. The method quantifies the level of HA-specific IgA antibodies relative to the total IgA content present in the sample. The assay employs an ELISA starter accessory kit from Bethyl Laboratories (Montgomery, TX, USA), including the microtiter plates, wash solution, blocking solution, sample/conjugate solution, coating solution, and 3,3′,5,5′-tetramethylbenzidine (TMB) solution. Briefly, ELISA plates are incubated overnight at 4 °C with 100 µL of HA antigen from A/California/07/2009 solution (H1N1) at 0.1 µg/well in coating buffer. After blocking and washing, nasopharyngeal samples and IgA standards were added to the plate and incubated for 2 h at 37 °C. After washing, goat anti-human IgA HRP conjugated antibody (Bethyl Laboratories) was added, followed by incubation for 1 hour at 37 °C. After additional washing steps, 100 µL of TMB substrate was added to the well to allow the enzymatic colour reaction to develop at room temperature (20–25 °C) in the dark for 18 min. The reaction was stopped by adding 100 μL of ELISA Stop Solution (0.18 M H_2_SO_4_) before a read-out in a microtiter plate reader at 450 nm. The anti-HA IgA concentration was extrapolated from a standard regression curve derived by diluting a human total IgA reference standard of established concentration. The value of influenza-specific IgA was finally normalized to the total IgA content and expressed as [influenza-specific IgA (U/mL)/total IgA (µg/mL) × 100].

### 2.9. Ad5 Neutralizing Antibody

The assessment of Ad5 neutralizing antibody in human sera was performed using a validated assay performed at Battelle Biomedical Research Center (West Jefferson, OH, USA). Dilutions of heat-inactivated human sera or control serum samples were mixed with an Ad5 vector that expresses green fluorescent protein (Ad5.CMV-GFP) at a final concentration of 3 × 10^6^ infectious units per mL and incubated at 37 °C for 60 ± 10 min to allow for neutralization of the virus. The mixtures were then inoculated onto Vero E6 cells in a 96-well plate and incubated for approximately three days. During this incubation, Ad5.CMV-GFP that was not neutralized would infect the cells and produce GFP, which was subsequently quantified using a microplate fluorimeter. The intensity of the GFP fluorescence was compared between test samples and controls inoculated with virus only or no virus to determine the level of neutralization that occurred. The reportable value for the assay was the MN50 value, corresponding to the reciprocal of the serum dilution that results in neutralization of 50% of the input Ad5.CMV-GFP. Values below the assay limit of quantification of 225 were assigned a value of 112.5.

### 2.10. Adenoviral Vector Shedding 

The assessment of adenoviral vector shedding in nasal samples was performed by MPI Research (Mattawan, MI, USA) according to a validated quantitative polymerase chain reaction (qPCR) assay. The assay was designed to differentiate between wild-type Ad5 and the replication-deficient NasoVAX vector. Following DNA extraction from nasopharyngeal swab samples, samples were diluted with 10 mM Tris buffer to a final DNA concentration of 100 ng/20 μL and stored at −80 °C until analysis. qPCR was performed on a 7900HT Fast Real Time PCR System fitted with a standard 96-well block. Samples, standards, and controls were tested in triplicates. qPCR reactions were prepared by mixing the diluted samples, standard, and controls with TaqMan Master Mix II (Thermo Fisher, Waltham, MA, USA), forward and reverse primers, probe, and nuclease free-water. The amount of vector DNA was quantified against a standard curve of plasmid vector DNA and expressed as copies of vector DNA per µg of genomic DNA in the sample.

### 2.11. Statistical Analysis 

Safety endpoints were analyzed descriptively in the Safety Population, defined as all subjects who provided informed consent, were randomized, and received investigational product. With 45 subjects receiving NasoVAX, the study had an 80% probability of detecting at least 1 AE that occurs at a 3.6% rate. If no SAEs were observed among the 45 subjects who received NasoVAX, an approximation to the 1-sided upper bound of the 90% confidence interval (CI) on the SAE occurrence rate would be 5%.

In addition, Ad5 shedding duration (days between vaccination and the last day with a positive test) was calculated for subjects in the Safety Population with shedding. Immunogenicity endpoints were analyzed in the Per-protocol Population, defined as all subjects in the Safety Population that received the assigned dose in accordance with the protocol, had HAI assay results predose and at Day 29, and had no major protocol deviations affecting the primary immunogenicity outcomes.

Immunogenicity results from the subjects enrolled in this study were compared to those from 20 similar subjects administered Fluzone in an earlier study conducted at the same research unit. Assay data were tabulated by visit using the applicable endpoint(s) and 95% CIs. Analysis of covariance (ANCOVA) was performed for antibody titer at each postbaseline visit, with log-transformed antibody titer as dependent variable, dose group as factor, and baseline log-transformed level as covariate. Comparisons of postbaseline log-transformed antibody titer were conducted for each NasoVAX dose group against the placebo group and against the Fluzone group, if applicable. From ANCOVA, least squares (LS) means and 95% CIs of the LS means, difference of LS means, and 95% CIs were obtained. Back-transforming the difference of LS mean estimates and their 95% CIs to the original scale resulted in a ratio of the geometric means. A post hoc analysis of median change from baseline to Day 8 for ELISpot SFU was added to best evaluate the skewed data. Categorical data (SPR, SCR, responder rate) were tabulated by counts, percentages, and 95% Clopper-Pearson CIs. Comparisons of responders in each NasoVAX dose group against the placebo group and the Fluzone group were conducted using Fisher’s exact test. Post hoc subgroup analyses by baseline Ad5 serostatus (positive [defined titer ≥ lower limit of quantitation] or negative) of Day 29 HAI assay GMR, Day 29 MN assay GMR, median Day 8 change from baseline in ELISpot SFU, and Day 29 IgA GMR were performed. All statistical tabulations and analyses were done using the SAS Version 9.4 or higher. Graphical representations were performed using R.

## 3. Results

### 3.1. Study Population 

**Figure 1:**
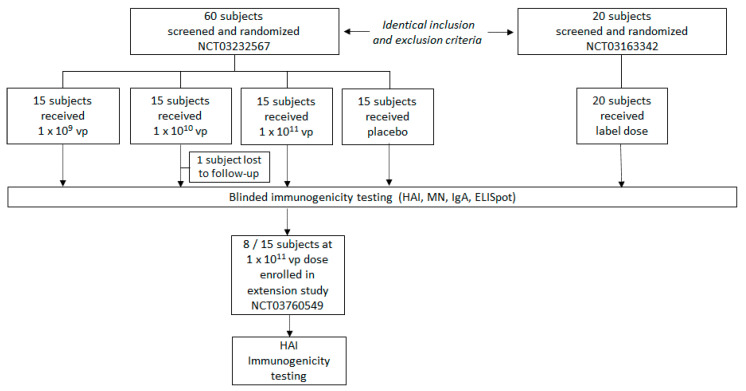
CONSORT Diagram. One subject in 1 × 10^10^ vp cohort lost to follow-up after D91. HAI = hemagglutination inhibition; MN = microneutralization; vp = viral particles.

Sixty subjects met the eligibility criteria and were enrolled and randomized to study drug or placebo (see Figure 1 for CONSORT diagram). The groups were well-balanced in age and race but more varied in sex distribution. No statistical differences were observed in the measures of baseline immunogenicity (Table 1). All 60 subjects received their assigned dose of study drug and were included in both the Safety and Per-protocol Populations; all completed the study except 1 subject in the 1 × 10^10^ vp group who withdrew because of relocation. Of the 15 subjects randomized to the 1 × 10^11^ vp NasoVAX group, 8 returned for immunogenicity analysis at a mean interval of 13.5 months postdose.

### 3.2. Safety 

NasoVAX was safe and well tolerated at single doses of 1 × 10^9^, 1 × 10^10^, and 1 × 10^11^ vp. No dose effect or difference between NasoVAX dose groups or overall NasoVAX group and the placebo group was seen for local or systemic reactogenicity events (Table 2), treatment-emergent AEs, or treatment-emergent AEs considered related to the investigational product (Table 3). The most common local reactions in the NasoVAX groups were nasal congestion, sneezing, nasal irritation, and sore throat. Notably, reported incidence of cough was similar in the NasoVAX and placebo groups. The most common systemic reactions in the NasoVAX groups were headache and fatigue; no subjects who received NasoVAX reported vomiting or fever. No subjects reported change in vision. All events were mild or moderate in severity except headache and fatigue for 1 subject each in the NasoVAX 1 × 10^9^ vp and 1 × 10^11^ vp groups, respectively. Reactogenicity event duration ranged from 1 to 14 days.

Shedding of the Ad5 vector inoculum was cleared from the nasopharynx in most subjects (86.7%) by Day 8 and in all subjects by Day 15 (Table S1). Median vector shedding duration was 2 days when including all subjects receiving NasoVAX, and also 2 days when including only subjects positive for shedding (data not shown). The number of subjects with any shedding increased with dose level at Days 4 and 8, but the magnitude of vector shedding was roughly equivalent across dose levels and represented insignificant fraction of the administered dose (less than 0.02% of the dose per mL of nasopharyngeal sample). No evidence of recombination of the NasoVAX vector with wild-type Ad5 was seen.

### 3.3. Immunogenicity

Baseline HAI and MN GMTs were generally similar across the placebo, Fluzone, and NasoVAX 1 × 10^9^ vp and 1 × 10^11^ vp groups but were higher in the NasoVAX 1 × 10^10^ vp group (Table 1). By contrast, the baseline geometric mean T cell immunity was lowest in the NasoVAX 1 × 10^10^ vp group.

**Figure 2:**
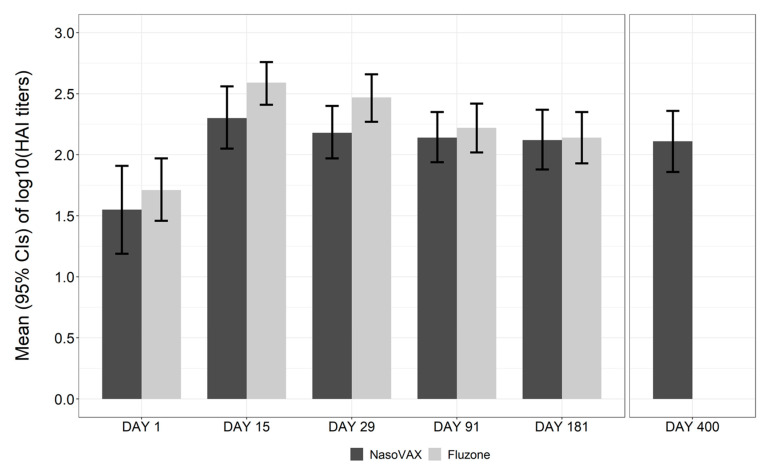
Durability of HAI Responses (1 × 10^11^ vp dose). The geometric mean titer ± 95% confidence interval of the HAI response for NasoVAX (1 × 10^11^ vp dose) and Fluzone against A/California/07/2009 (H1N1) are shown. The data are a mean of 15 subjects for NasoVAX through D181, 8 NasoVAX subjects for the D400data, and 20 subjects for Fluzone. CI = confidence interval; HAI = hemagglutination inhibition.

A NasoVAX dose trend for HAI GMT, GMR, SCR, and SPR and for MN GMT, GMR, and responder rates was observed (Table 4). Administration of NasoVAX at the mid and high doses resulted in a 100% seroprotection rate, consistent with the 95% seroprotection rate observed with Fluzone. The HAI GMT of all NasoVAX groups and the Fluzone group at Day 29 were statistically higher than the placebo group. The Day 29 HAI GMT and GMR results for the Fluzone group were numerically higher than in the NasoVAX groups, but the difference was not statistically significant compared to the mid- and high-dose NasoVAX group. The durability of the HAI titer for the 1 × 10^11^ vp NasoVAX group was stable between Day 29 and Day 181, while the response to Fluzone declined approximately 50% over the same period (Figure 2), though the titers on Day 181 were comparable with NasoVAX (Figure 2).

Microneutralization titers at Day 29 for the high dose of NasoVAX and Fluzone against the represented influenza strain were essentially the same. NasoVAX HAI and MN titers against divergent influenza strains and subtypes at Day 29 were low or absent and similar to Fluzone (data not shown). 

Eight of the 15 subjects that completed the study in the NasoVAX 1 × 10^11^ vp dose cohort returned between 10 and 14 months postdose (mean 13.5 months) for HAI analysis. Following a single intranasal administration of NasoVAX, the HAI titer response was maintained essentially unchanged from Day 29 to the final analysis 10 to 14 months postdose (Figure 2).

**Figure 3:**
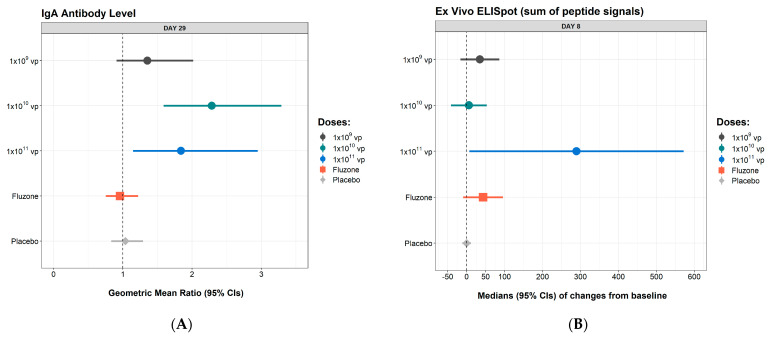
HA-specific Mucosal IgA and T Cell Immune Responses. Nasal mucosal and cellular immunes responses to HA are shown. (**A**). Geometric mean ratio ± 95% confidence interval of the anti-A/California/07/2009 (H1N1) IgA responses at Day 29 for all subjects are shown. (**B**). Median change from baseline of the IFN-secreting ELISpot responses against HA of A/California/04/2009 (H1N1) at Day 8 are shown (sum of all HA peptides responses) at D8 are shown. CI = confidence interval; vp = viral particles. Plotted are data from all subjects except one subject each in the mid- and high-dose groups with missing values. CI = confidence interval.

NasoVAX elicited a mucosal IgA immune response specific to influenza A/California/07/2009 (H1N1) not seen in either the placebo or Fluzone groups (Figure 3A). Within the NasoVAX dose groups, 53% (8/15) of subjects in the low-dose group, and 87% (13/15) of subjects in the mid- and high-dose groups had an increase in IgA following vaccination. NasoVAX 1 × 10^10^ vp and 1 × 10^11^ vp dose groups rose 2.3- and 1.8-fold, respectively, over baseline and were significantly higher than placebo or Fluzone at Day 29 (each *p* ≤ 0.05), indicating that NasoVAX was able to elicit a local mucosal immune response following intranasal administration.

NasoVAX also elicited HA-specific T cell responses as measured by an *ex vivo* IFN-γ ELIspot. The number of subjects with at least a 100-spot forming cell count increase over their baseline value was 3/15, 3/15 and 11/15 for the low-, mid- and high-dose groups resectively. The median change in SFU per million PBMCs from baseline to Day 8 showed larger increases in the NasoVAX groups than in the placebo group, reaching significance (*p* ≤ 0.05) in the 1 × 10^11^ vp group (Figure 3B). The median increase in ELISpot SFU in the 1 × 10^11^ vp dose group was approximately 7-fold greater than in the Fluzone group. There was no clear vaccine dose effect on the T cell response, as the low- and mid-dose of NasoVAX were associated with only minor T cell responses.

Predose serum anti-Ad5 immunity was roughly comparable across each of the NasoVAX groups with the median titer of anti-Ad5 neutralizing antibody in seropositive individuals 29- to 45-fold greater than in seronegative individuals. An analysis of the HAI, MN, T cell, and IgA response results by baseline Ad5 serostatus showed that at lower NasoVAX doses, pre-existing Ad5 immunity appeared to have varying effects on the immune response, with humoral responses moderately affected (Table 5). No suppression of HAI, MN, T cell, and mucosal immunogenicity was observed in the NasoVAX high dose group. Mucosal IgA responses appeared to be less sensitive to the suppressive effects of pre-existing vector immunity with equal or greater IgA responses in the seropositive subjects compared to seronegative subjects, while the effect against cellular immunity, especially at lower NasoVAX dose levels, appeared to be more pronounced.

## 4. Discussion

A single intranasal dose of NasoVAX was well-tolerated and elicited a broad immune response that included humoral, cellular, and mucosal responses with HAI responses that were durable for at least one year on average. These results identify NasoVAX as an influenza vaccine possessing key attributes of an improved seasonal influenza vaccine as recently discussed by NIAID [11].

In this study, rates of local and systemic reactogenicity and AEs observed with NasoVAX at doses up to 1 × 10^11^ vp were comparable to saline placebo, demonstrating a safety profile appropriate for its intended use as a prophylactic vaccine in the general population. In contrast to the licensed intranasal live attenuated influenza vaccine (LAIV), NasoVAX is unable to replicate following administration and, as such, has the potential to exhibit an improved safety profile compared to the licensed LAIV vaccine in populations with immune systems that are either incompletely developed or compromised by age or other factors.

Participants receiving either of the two highest NasoVAX doses achieved 100% seroprotection at Day 29. Point estimates of HAI GMT or seroconversion rates at the most immunogenic dose of NasoVAX (1 × 10^11^ vp) were somewhat lower than Fluzone, but the difference was not statistically significant. Importantly, the HAI response to NasoVAX appeared more durable than the response to Fluzone with NasoVAX HAI titers remaining stable throughout the 6-month per protocol evaluation period, and ultimately to more than 1-year on average as documented in the NasoVAX extension study. In contrast, HAI titers to Fluzone declined by approximately 50% from their peak level over the 6 months but remained comparable to NasoVAX titers at the end of the 6 months per protocol evaluation period. Microneutralization titers against the influenza strain represented in the vaccine for NasoVAX were approximately equal to those of Fluzone over the 6-month study period.

The induction of a mucosal immune response in the nasopharynx following administration with NasoVAX is consistent with the intranasal route of delivery, as the development of mucosal immunity requires antigen presentation to occur in a mucosal environment [18]. Intranasal vaccination with LAIV is associated with a mucosal IgA response in the nasopharyngeal cavity of similar magnitude as NasoVAX [3,5], and human influenza challenge studies with intranasally administered LAIV has shown that inductions of IgA similar to the magnitude observed in this study are associated with protection from influenza disease [3,4]. Likely due to the ability of intranasal dosing to induce mucosal immunity, LAIV has also been shown to provide better protection against the transmission of influenza virus than an intramuscularly administered inactivated influenza virus vaccine [19], a quality that could be of significant importance in the control of a pandemic.

Along with serum and mucosal antibody responses, T cell immunity has been associated with prevention of influenza disease and lessening of symptoms or duration of illness [7,8], but T cell responses to inactivated influenza vaccines are typically low in the absence of an adjuvant. Following administration of NasoVAX, the vector infects epithelial cells of the nasal cavity and upper respiratory tract, where the antigen is expressed in the absence of viral replication. The expressed antigen can then be presented to the immune system through the HLA class I antigen processing machinery, leading to antiviral CD8+ T cell responses as observed in other studies [20]. The substantially greater T cell response associated with NasoVAX compared to the inactivated influenza vaccine comparator is consistent with the potential for Ad5 vectors to activate a systemic cellular immune response greater than other human adenovirus serotype vaccines [21]. It is unclear why the T cell response in the 1 × 10^10^ vp dose group was appreciably lower than that noted in the 1 × 10^11^ vp dose group, especially in light of the higher baseline influenza immunity in the 1 × 10^10^ vp dose group. It is possible there may be a threshold for immune activation required to stimulate T cell responses, but additional studies will likely be required to understand the cellular response to NasoVAX. While not evaluated in this study, mucosal administration of Ad5 vectors promotes the establishment of resident memory T cells in preclinical models [20], a cell population of emerging importance in protection against invading pathogens, especially in viral infection [22,23].

Between 50–90% of the global population are seropositive for Ad5, depending on the geographical region [24]. The ability of pre-existing Ad5 immunity to suppress the functionality of Ad5-based vectors has been well-documented [25] and has led to the development of alternative adenoviral vector serotypes with lower rates of seroprevalence in the population. Nearly all replication-deficient Ad5 vector studies showing suppressive effects of vector seropositivity were performed using parenteral routes of administration. However, several studies have suggested that when administered by the intranasal route, Ad5-based vaccines can surmount pre-existing anti-vector immunity and result in robust immune responses [16,26,27]. The data presented in this study confirm earlier preclinical studies using intranasal administration and demonstrate that intranasal administration can achieve similar immune responses in participants that were either seronegative or seropositive for Ad5 prior to NasoVAX administration. NasoVAX is based on a replication-deficient Ad5 vaccine platform technology that is ideally suited for not only rapid development of vaccines, as necessary to meet the evolving strain requirements of a seasonal influenza vaccine, but also valuable in the event of a respiratory virus pandemic, such as the current pandemic caused by severe acute respiratory syndrome virus coronavirus 2 (SARS-CoV-2). The vaccine attributes described for NasoVAX in this study are likely to translate to other intranasal vaccine candidates based on the same platform technology [20].

## 5. Conclusions

A single intranasal dose of NasoVAX was well-tolerated and induced a broad immune response that engaged multiple arms of the adaptive immune response, including systemic antibody and T cell immunity, as well as a local mucosal antibody response in the respiratory tract, in both seronegative and seropositive participants. The vaccine-induced immune response mirrored the natural immune response to influenza infection and may provide better vaccine protection against disease than current influenza vaccines. Moreover, the development of a local mucosal response in the respiratory tract may have important implications for the control of influenza transmission and the spread of infection. Future studies will include evaluation of a quadrivalent formulation.

## Figures and Tables

**Table 1:** Demographic and Baseline Characteristics.

Characteristic	NasoVAX			Placebo *N* = 15	Fluzone *N* = 20
	1 × 10^9^ vp *N* = 15	1 × 10^10^ vp *N* = 15	1 × 10^11^ vp *N* = 15		
**Sex, *n* (%)**					
Female	11 (73.3)	10 (66.7)	6 (40.0)	6 (40.0)	10 (50.0)
Male	4 (26.7)	5 (33.3)	9 (60.0)	9 (60.0)	10 (50.0)
**Age (years)**					
Mean (SD)	30.1 (8.50)	31.6 (8.54)	32.6 (9.35)	29.3 (6.87)	34.2 (8.69)
Range: minimum, maximum	19, 47	18, 46	21, 48	20, 45	21, 49
**Race, *n* (%) ^a^**					
White	8 (53.3)	9 (60.0)	8 (53.3)	9 (60.0)	9 (45.0)
Black	6 (40.0)	6 (40.0)	6 (40.0)	6 (40.0)	9 (45.0)
Asian	1 (6.7)	2 (13.3)	2 (13.3)	0	0
Other	1 (6.7)	0	0	0	2 (10.0)
**Immunogenicity ^b^**					
HAI GMT (95% CI)	39.1 (19.4, 78.8)	71.3 (32.6, 156.0)	35.6 (15.6, 81.4)	30.3 (14.0, 65.8)	51.9 (28.9, 93.2)
Microneutralization GMT (95% CI)	21.4 (10.5, 43.7)	44.9 (21.5, 93.6)	27.6 (13.3, 57.4)	16.2 (8.5, 31.2)	26.4 (14.5, 48.2)
ELISpot GM SFU/10^6^ cells (95% CI)	3.6 (1.2, 11.0)	1.8 (0.7, 4.4)	6.9 (2.0, 24.2)	7.2 (1.8. 28.4)	2.9 (1.3, 6.3)

Abbreviations: CI = confidence interval; GM = geometric mean; GMT = geometric mean titer; HAI = hemagglutination inhibition; SD = standard deviation; SFU = spot-forming units. ^a^: Subjects may have reported more than 1 race. ^b^: A/California/07/2009(H1N1), except ELISpot which was A/California/04/2009(H1N1).

**Table 2:** Local and Systemic Solicitated Adverse Events by Dose Group—Safety Population.

Event	NasoVAX				Placebo *N* = 15 *n* (%)
	1 × 10^9^ vp *N* = 15 *n* (%)	1 × 10^10^ vp *N* = 15 *n* (%)	1 × 10^11^ vp *N* = 15 *n* (%)	Overall *N* = 45 *n* (%)	
**Any local or systemic event**	**13 (86.7)**	**12 (80.0)**	**8 (53.3)**	**33 (73.3)**	**7 (46.6)**
**Any local event**	**9 (60.0)**	**9 (60.0)**	**6 (40.0)**	**24 (53.3)**	**6 (40.0)**
Nasal irritation	3 (20.0)	5 (33.3)	1 (6.7)	9 (20.0)	2 (13.3)
Sneezing	4 (26.7)	5 (33.3)	3 (20.0)	12 (26.7)	3 (20.0)
Nasal congestion	6 (40.0)	7 (46.7)	2 (13.3)	15 (33.3)	4 (26.7)
Cough	3 (20.0)	1 (6.7)	2 (13.3)	6 (13.3)	3 (20.0)
Sore throat	4 (26.7)	4 (26.7)	3 (20.0)	11 (24.4)	3 (20.0)
Change in smell	2 (13.3)	0	0	2 (4.4)	1 (6.7)
Change in taste	1 (6.7)	1 (6.7)	0	2 (4.4)	2 (13.3)
Change in vision	0	0	0	0	0
Eye pain	2 (13.3)	3 (20.0)	0	5 (11.1)	1 (6.7)
**Any systemic event**	**9 (60.0)**	**11 (73.3)**	**5 (33.3)**	**25 (55.6)**	**6 (40.0)**
Headache	7 (46.7)	10 (66.7)	4 (26.7)	21 (46.7)	6 (40.0)
Fatigue	5 (33.3)	7 (46.7)	3 (20.0)	15 (33.3)	4 (26.7)
Muscle ache	2 (13.3)	1 (6.7)	3 (20.0)	6 (13.3)	1 (6.7)
Nausea	2 (13.3)	2 (13.3)	0	4 (8.9)	2 (13.3)
Vomiting	0	0	0	0	1 (6.7)
Diarrhea	0	0	1 (6.7)	1 (2.2)	1 (6.7)
Chills	0	1 (6.7)	0	1 (2.2)	0
Fever	0	0	0	0	0

Abbreviations: vp = viral particles.

**Table 3:** Number of Subjects Reporting Vaccine-related Adverse Events.

Preferred Term	NasoVAX				Placebo *N* = 15 *n* (%)
	1 × 10^9^ vp *N* = 15 *n* (%)	1 × 10^10^ vp *N* = 15 *n* (%)	1 × 10^11^ vp *N* = 15 *n* (%)	Overall *N* = 45 *n* (%)	
Any adverse event	2 (13.3)	5 (33.3)	3 (20.0)	10 (22.2)	3 (20.0)
Skin abrasion ^a^	1 (6.7)	1 (6.7)	2 (13.3)	4 (8.9)	1 (6.7)
Epistaxis	0	0	2 (13.3)	2 (4.4)	0
Rhinorrhoea	1 (6.7)	1 (6.7)	0	2 (4.4)	0
Chills	0	0	1 (6.7)	1 (2.2)	0
Ear pain	0	0	1 (6.7)	1 (2.2)	0
Flushing	0	0	1 (6.7)	1 (2.2)	0
Laryngitis	0	1 (6.7)	0	1 (2.2)	0
Sinusitis	0	1 (6.7)	0	1 (2.2)	0
Upper respiratory tract infection	0	1 (6.7)	0	1 (2.2)	0
Lacrimation increased	0	0	0	0	1 (6.7)
Nasal septum ulceration	0	0	0	0	1 (6.7)

^a^ Verbatim terms indicates location as intranasal or nares.

**Table 4:** HAI and MN Responses by Treatment Group Four Weeks Post-Vaccination.

Statistic	NasoVAX			Placebo	Fluzone
	1 × 10^9^ vp	1 × 10^10^ vp	1 × 10^11^ vp		
**Hemagglutinin Inhibition Response at Day 29**					
GMT (95% CI) ^a^	**83.8** **(42.1, 166.7)**	**160.0** **(94.8, 270.0)**	**152.8** **(93.5, 249.7)**	27.6 (13.5, 56.8)	**293.4** **(186.7, 461.3)**
GMR (95% CI)	2.1 (1.1, 4.2)	2.2 (1.2, 4.3)	**4.3** **(1.5, 12.0)**	0.9 (0.7, 1.2)	**5.7** **(2.9, 11.2)**
SCR ^b^ (95% CI)	13.3% (1.7%, 40.5%)	26.7% (7.8%, 55.1%)	**33.3%** **(11.8%, 61.6%)**	0.0% (0.0%, 21.8%)	**50.0%** **(27.2%, 72.8%)**
SPR ^c^ (95% CI)	80.0% (51.9%, 95.7%)	**100.0%** **(78.2%, 100.0%)**	**100.0%** **(78.2%, 100.0%)**	53.3% (26.6%, 78.7%)	**95.0%** **(75.1%, 99.9%)**
**Microneutralization Response at Day 29**					
GMT (95% CI)	44.9 (21.8, 92.3)	**113.1** **(58.0, 220.8)**	**142.5** **(93.6, 217.1)**	17.8 (9.1, 35.0)	**162.8** **(95.8, 276.6)**
Responder rate 4-fold rise (95% CI)	13.3% (1.7%, 40.5%)	26.7% (7.8%, 55.1%)	**53.3%** **(26.6%, 78.7%)**	0.0% (0.0%, 21.8%)	**50.0%** **(27.2%, 72.8%)**

Bold values are statistically significantly higher than values in placebo group (*p* ≤ 0.05). Abbreviations: CI = confidence interval; GMR = geometric mean ratio; GMT = geometric mean titer; HAI = hemagglutination inhibition; LS = least squares; SCR = seroconversion rate; SFU = spot-forming units; SPR = seroprotection rate; vp = virus particles. ^a^ The analysis of covariance uses log-transformed level as dependent variable, dose group as a factor, and baseline log-transformed analysis as a covariate. Differences of LS mean estimates and 95% CIs were back-transformed to the original scale, resulting in a ratio of the geometric means. ^b^ The percentage of subjects with either a baseline HAI titer < 1:10 and a postvaccination titer ≥ 1:40 (which is 4 times the assay lower limit of quantitation), or a baseline HAI titer ≥ 1:10 and a 4-fold increase in postvaccination HAI titer relative to baseline. ^c^ The percentage of subjects with a HAI titer ≥ 1:40.

**Table 5:** Effect of Pre-existing Adenovirus Serotype 5 Status on NasoVAX Immunogenicity.

Immunogenicity Endpoint	NasoVAX					
	1 × 10^9^ vp		1 × 10^10^ vp		1 × 10^11^ vp	
Predose Ad5 Serostatus	Positive ^a^ *N* = 7	Negative *N* = 8	Positive ^a^ *N* = 8	Negative *N* = 7	Positive ^a^ *N* = 10	Negative *N* = 5
Predose Anti-Ad5 Neutralizing Titer GMT (95% CI)	5110.7 (1024.9, 25,484.7)	112.4 (112.4, 112.4)	3250.1 (827.5, 12764.4)	112.4 (112.4, 112.4)	4015.4 (1483.7, 10866.9)	112.4 (112.4, 112.4)
**Immunogenicity Endpoint**						
Day 29 HAI GMR (95% CI)	1.22 (0.89,1.67)	3.51 (1.02, 12.12)	1.61 (0.70, 3.71)	3.28 (0.95, 11.33)	4.44 (1.28, 15.38)	4.00 (0.23, 69.46)
Day 29 MN GMR (95% CI)	1.22 (0.89, 1.67)	3.36 (0.88, 12.86)	1.61 (0.84, 3.10)	4.20 (1.21, 14.63)	5.46 (1.83, 16.30)	4.60 (0.58, 36.68)
Day 8 change from baseline (SFU/106 cells), median (95% CI)	0 (−17.2, 17.2)	89 (−21.5, 198.5)	0 (−48.1, 48.1)	43 (−26.8, 112.8)	267 (46.8, 487.2)	735 (323.6,1146.4)
Day 29 IgA GMR (95% CI)	1.03 (0.79, 1.35)	1.72 (0.81, 3.68)	2.62 (1.64, 4.16)	1.96 (0.96, 4.00)	1.87 (1.01, 3.44)	1.78 (0.55, 5.77)
Day 29 anti-Ad5 GMR (95% CI)	1.09 (0.83, 1.44)	2.43 (0.84, 7.05)	1.42 (0.78, 2.56)	1.79 (0.89, 3.61)	2.47 (1.25, 4.89)	2.10 (0.53, 8.38)

Abbreviations: Ad5 = adenovirus serotype 5; CI = confidence interval; GMR = geometric mean ratio; HAI = hemagglutination inhibition; IgA = immunoglobulin A; LLOQ = lower limit of quantitation; SFU = spot-forming units. ^a^ Ad5 seropositive defined as Ad5 neutralizing antibody titer ≥ LLOQ (225).

## Supplementary Materials

The following are available online at https://www.mdpi.com/2076-393X/9/3/224/s1, Table S1: Adenovirus Serotype 5 Vector Shedding by Dose Group—Safety Population.
