# Supplementary material for: Safety and Immunogenicity of a Novel Intranasal Influenza Vaccine (NasoVAX): A Phase 2 Randomized, Controlled Trial
Source: Vaccines (Basel). 2021 Mar 5;9(3):224. doi: 10.3390/vaccines9030224 (PMC8000446; doi:10.3390/vaccines9030224)
Supplement: Supplementary file 1 [file vaccines-09-00224-s001.pdf]

Table S1. Adenovirus Serotype 5 Vector Shedding by Dose Group – Safety Population.

	NasoVAX				Placebo $N = 15$ Copies/5 µL
	**1 × 10^9 vp** $N = 15$ Copies/5 µL	**1 × 10^{10} vp** $N = 15$ Copies/5 µL	**1 × 10^{11} vp** $N = 15$ Copies/5 µL	**Overall** $N = 45$ Copies/5 µL	
**Day 4**					
Subjects with shedding, *n* (%)	7 (46.7)	9 (60.0)	12 (80.0)	28 (62.2)	0
Log_{10} copies/5 µL, median (range)	4.0 (1.4, 4.3)	3.8 (1.4, 5.4)	4.4 (1.4, 6.3)	4.2 (1.4, 6.3)	NA
**Day 8**					
Subjects with shedding , *n* (%)	1 (6.7)	2 (13.3)	3 (20.0)	6 (13.3)	0
Log_{10} copies/5 µL, median (range)	4.3 (4.3, 4.3)	1.4 (1.4, 1.4)	4.0 (1.4, 4.0)	2.7 (1.4, 4.3)	NA
**Day 15**					
Subjects with shedding, *n* (%)	0	0	0	0	0
**Duration (Days)**					
Median (range)	0.0 (0, 7)	2.0 (0, 6)	2.0 (0, 7)	2.0 (0, 7)	0.0 (0, 0)

Abbreviations: NA = not applicable; vp = viral particles.
